# Disparities and Medical Expenditure Implications in Pediatric Tele-Mental Health Services During the COVID-19 Pandemic in Mississippi

**DOI:** 10.1007/s11414-024-09906-2

**Published:** 2024-08-21

**Authors:** Yunxi Zhang, Lincy S. Lal, Yueh-Yun Lin, J. Michael Swint, Ying Zhang, Richard L. Summers, Barbara F. Jones, Saurabh Chandra, Mark E. Ladner

**Affiliations:** 1 Department of Data Science, University of Mississippi Medical Center, Jackson, MS USA; 2 Center for Telehealth, University of Mississippi Medical Center, Jackson, MS USA; 3 Department of Management, Policy and Community Health, The University of Texas School of Public Health, Houston, TX USA; 4 John P and Katherine G McGovern Medical School, Institute for Clinical Research and Learning Healthcare, The University of Texas Health Science Center at Houston, Houston, TX USA; 5 Center for Informatics and Analytics, University of Mississippi Medical Center, Jackson, MS USA; 6 Department of Emergency Medicine, University of Mississippi Medical Center, Jackson, MS USA; 7 Department of Psychiatry and Human Behavior, University of Mississippi Medical Center, Jackson, MS USA; 8 Department of Medicine, University of Mississippi Medical Center, Jackson, MS USA

## Abstract

Tele-mental health (TMH) services, including both mental and behavioral healthcare (MBH) services, emerged as a cornerstone in delivering pediatric mental healthcare during the coronavirus disease 2019 (COVID-19) pandemic, yet their utilization and effects on healthcare resource utilization (HCRU) and medical expenditures remain unclear. To bridge the gap, this study aims to investigate the association between TMH utilization and sociodemographic factors and assess its associated HCRU and medical expenditures within a pediatric population in Mississippi. Studying 1,972 insured pediatric patients who accessed outpatient MBH services at the study institution between January 2020 and June 2023, age, race, insurance type, rural residency, and household income were identified as key determinants of TMH utilization. Adjusting for sociodemographics, TMH utilization was associated with 122% more MBH-associated outpatient visits and 36% higher related medical expenditures, but 27% less overall medical expenditures. This study reveals sociodemographic disparities in pediatric TMH utilization, highlights its role in augmenting outpatient mental healthcare access, and shows its potential for cost savings. Future efforts should aim at fostering more digitally inclusive, equitable, and affordable pediatric mental healthcare services.

## Introduction

Amid the tumultuous landscape of the coronavirus disease 2019 (COVID-19) pandemic, the United States (U.S.) healthcare system underwent transformative changes that altered how healthcare services were delivered.^1^ During outbreak periods, the exigency of social distancing catalyzed a shift towards telehealth as a vital alternative to traditional in-person care.^2^ This shift was particularly notable in mental health, where tele-mental health (TMH), a form of virtual psychiatric and psychological care for mental and behavioral health, gained widespread adoption. Particularly, amid school closures and ensuing emotional and psychological disruptions, TMH emerged as a critical resource for the vulnerable demographic of children and adolescents.^3^ With subsequent policy changes and the easing of the COVID-19 spread and restrictions, TMH has evolved into an essential adjunct to in-person care services.^4,5^

Youth mental and behavioral health remains a pressing concern in the U.S., with more than 2.7 million youths grappling with severe major depression in 2023.^6^ Compounding this situation is the scarcity of child and adolescent psychiatrists, with nearly 80% of U.S. areas facing glaring shortages.^7,8^ In 2021, a national emergency in child mental and behavioral health was declared due to the exacerbated crisis worsened by COVID-19 and entrenched systemic disparities.^9^ While TMH has been hailed as a potential solution for improving access to care, particularly in underserved areas, its rapid adoption also raised questions about its role in promoting health equity and its economic implications to payers. A previous study conducted among the Mississippi Medicaid population revealed that TMH uptake was associated with increasing age and was more prevalent among non-Hispanic White individuals, despite the narrowing of the digital divide.^10,11^ Furthermore, previous studies indicate that TMH was associated with cost savings among Medicare populations,^10,12^ did not significantly affect total healthcare costs among commercially insured populations before COVID-19,^13^ but was associated with higher costs among the Mississippi Medicaid population between 2019—2020.^10^ The unprecedented rise in TMH usage provides a ground for scrutinizing its utilization across varied sociodemographic groups, as well as its implications for healthcare resource utilization (HCRU) and medical expenditures.

Mississippi is predominantly rural and economically disadvantaged, with children of color experiencing disproportionately high poverty rates.^14^ During the COVID-19 pandemic, the Shelter-In-Place order in Mississippi requires all non-essential business and operations to cease except for performing minimum operations and limits non-essential travel.^15^ The state governor also requested statewide school closure through April 17, 2020.^16^ The University of Mississippi Medical Center (UMMC), Mississippi’s only academic medical center, located in the Jackson metropolitan area and serving a diverse patient population including those from rural and underserved areas, stands as a microcosm of systemic health inequities. Though TMH services existed at UMMC before the pandemic, the demand for them surged, especially during the COVID-19 outbreak.^17^ Following the declaration of the COVID-19 pandemic, UMMC and its affiliated sites transitioned most mental and behavioral health services to TMH within a week.^18^ Previous research at UMMC underscored the ongoing necessity for accessible outpatient services for patients of all sociodemographic groups and suggested the use of telehealth for mental and behavioral health management.^19^ Nevertheless, patterns of pediatric TMH utilization among different sociodemographic groups remain inadequately understood.

To bridge this knowledge gap, this study aims to investigate the association between TMH utilization and sociodemographic factors, as well as assess its associated HCRU and medical expenditures within a pediatric population in Mississippi. Leveraging the diversity of the patient population at UMMC, this study provides insights into TMH equity in the pediatric healthcare context of Mississippi in terms of the key sociodemographic determinants of TMH utilization along with the increased mental and behavioral disorder-related outpatient and medical expenditures, but the decreased all-cause medical expenditures.

## Methods

This study received approval from the Institutional Review Board at UMMC with a waiver of informed consent. The report adheres to the Strengthening the Reporting of Observational Studies in Epidemiology (STROBE) guideline.^20^

### Study design and participants

A retrospective cohort study was conducted focusing on children and adolescents (age < 18 years) who accessed mental and behavioral health outpatient services from the Department of Psychiatry and Human Behavior at UMMC. The study spanned from January 1, 2020, to June 30, 2023. Using electronic health records, mental and behavioral health visits were identified based on the first two diagnosis codes, with either falling in the F01 – F99 range of the International Classification of Diseases, Tenth Revision (ICD-10). The first mental and behavioral health visit during the study period was considered the baseline for each patient. For a comprehensive assessment of HCRU and medical expenditures, patients who were uninsured or did not consistently seek any healthcare at UMMC were excluded, as their records at UMMC may not be representative of the complete and exhaustive HCRU and medical expenditures. Specifically, the criteria for the consistently seeking healthcare at UMMC required that patients had (1) scheduled at least three visits per year for two years and (2) completed a minimum of two outpatient visits with a gap of at least 3 months between them, all within the study timeframe for any medical reasons. These criteria were developed based on previous studies conducted in an academic tertiary care setting.^21^ After selecting the study participants, two cohorts were defined: one included individuals who had at least one TMH encounter (TMH cohort), and the other included those who did not have any TMH encounters (non-TMH cohort). TMH encounters were defined based on the visit type documented in UMMC electronic health records.

### Measures

Comparative analyses were conducted on sociodemographic characteristics, HCRU, and medical expenditures between the TMH and non-TMH cohorts. For HCRU, mental and behavioral health-associated and all-cause utilization were examined, encompassing metrics for outpatient visits, inpatient admissions, and Emergency Department (ED) visits. Correspondingly, the medical expenditures were calculated for both mental and behavioral health-associated and all-cause services. Due to the variability in insurance fee schedules, the calculation of medical expenditures was adjusted using Mississippi Medicaid fee schedules through Current Procedural Terminology (CPT). Specifically, fee schedules of the Outpatient Prospective Payment System (OPPS) for hospital services and mental health services in 2023 were applied.^22^ Given the variability in follow-up duration, HCRU and medical expenditures were reported on a per-patient-per-month (PPPM) basis.

Patient sociodemographic characteristics considered in the study include age, sex, race, primary insurance, rurality, and household income. Considering that TMH and healthcare resource utilizations may vary depending on patient developmental stages, including both psychological and physical aspects, age was treated as a categorical variable.^23,24^ Age categories were based on the first completed TMH visit during the study and were grouped as follows: birth to 5 years (early childhood), 6 to 12 years (middle childhood), and 13 to 17 years (adolescence). Race was categorized into three groups: White/Caucasian, Black/African American, and others. A small number of patients (2.8%) had unknown or unreported race, which were considered missing and excluded from the analysis. Primary insurance was determined by the most frequently used insurance for mental and behavioral health visits and categorized into commercial insurance, Medicaid, and others, such as workers’ compensation and managed care. Rurality was determined using the Rural–Urban Commuting Area Codes, with codes above 3 indicating rural areas. Annual household income was estimated using the median data of each county from the U.S. Census Bureau’s Small Area Income and Poverty Estimates program.^25^

### Statistical analysis

The association between sociodemographic factors and TMH utilization was investigated by calculating odds ratios (ORs) and their corresponding 95% confidence intervals (CIs), complemented by Pearson’s $\chi$^2^ tests. Additionally, the associations between TMH utilization and both HCRU and medical expenditures for mental and behavioral health services and all-cause utilization were evaluated using Wilcoxon rank-sum tests. To estimate the effects of TMH utilization on HCRU and medical expenditures, generalized linear regression models (GLMs) with log-link functions were constructed while adjusting for sociodemographic factors. Depending on the distribution of outcome variables, negative binomial regression models or zero-inflated Poisson regression models were fitted for HCRU variables, and Gamma regression models were fitted for medical expenditures. Two-sided tests with an alpha level of 0.05 were used to determine statistical significance. Available case analysis was used to deal with missing data.^26^ All analyses were performed using SAS statistical software (version 9.4, SAS Institute Inc., Cary, NC).

## Results

### Sociodemographic characteristics

**Figure 1:**
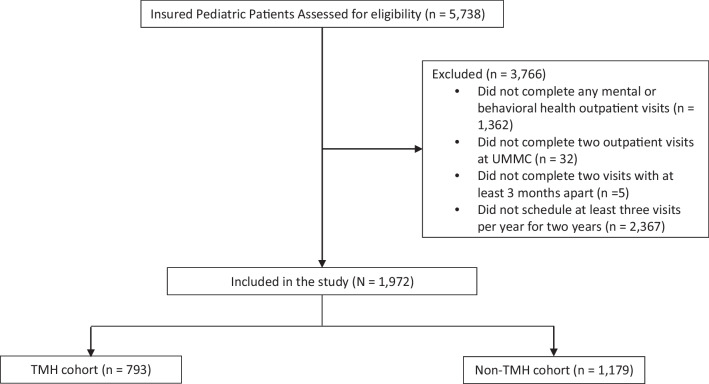
Flowchart of study participants selection

The study encompassed 1,972 pediatric patients. Of these, 793 utilized TMH services, while 1,179 did not (Fig. 1). Table 1 displays their sociodemographic characteristics. Overall, most patients fell into middle childhood (44.37%), were male (53.85%), identified as Black/African American (52.71%), were covered by Medicaid (66.68%), resided in urban areas (63.67%), and had annual household incomes ranging from $42,000 to $50,000 (43.46%).

**Table 1:** Baseline sociodemographic characteristics (*N* = 1972)

	All patients (*N* = 1972)	TMH (*n* = 793)	Non-TMH (*n* = 1179)	Odds ratio (95% CI)	*p* value
Age, no. (%)					< .001***
Early childhood	288 (14.60)	19 (2.40)	269 (22.82)	Ref	
Middle childhood	875 (44.37)	384 (48.42)	491 (41.65)	11.07 (6.82, 17.96)	
Adolescence	809 (41.02)	390 (49.18)	419 (35.54)	13.18 (8.11, 21.40)	
Female, no. (%)	910 (46.15)	371 (46.78)	539 (45.72)	1.04 (0.87, 1.25)	0.641
Race, no. (%)					< .001***
White/Caucasian	797 (41.60)	399 (52.57)	398 (34.40)	Ref	
Black/African American	1010 (52.71)	321 (42.29)	689 (59.55)	0.47 (0.38, 0.56)	
Others	109 (5.69)	39 (5.14)	70 (6.05)	0.56 (0.37, 0.84)	
Primary insurance, no. (%)					< .001***
Medicaid	1315 (66.68)	474 (59.77)	841 (71.33)	Ref	
Commercial	565 (28.65)	268 (33.80)	297 (25.19)	1.60 (1.31, 1.96)	
Other	92 (4.67)	51 (6.43)	41 (3.48)	2.21 (1.44, 3.38)	
Rural residency, no. (%)	716 (36.33)	248 (31.27)	468 (39.73)	0.69 (0.57, 0.84)	< .001***
Household income, $, no. (%)					< .001***
≤ 42,000	389 (19.73)	137 (17.28)	252 (21.37)	Ref	
42,000 – 50,000	857 (43.46)	296 (37.33)	561 (47.58)	0.97 (0.76, 1.25)	
> 50,000	726 (36.82)	360 (45.40)	366 (31.04)	1.81 (1.40, 2.33)	

Abbreviations: *TMH* Tele-Mental Health; *CI* Confidence interval

*p* values were calculated using the Pearson’s χ2 test

^*^ *p* < 0.05, ** *p* < 0.01, *** *p* < .001

Age, race, primary insurance, rural residency, and household income were significantly associated with TMH adoption (all *p* < 0.001), but not for sex. In the TMH cohort, adolescence comprised the largest age group (49.18%), while middle childhood accounted for 48.42%. This differed from the non-TMH cohort, where the largest age group was middle childhood (41.65%), followed by adolescence (35.54%). The TMH cohort had a much lower proportion of early childhood (2.40% vs 22.82%). Black/African American patients and patients of other races were less likely to use TMH services compared to White/Caucasian patients with ORs of 0.47 (95% CI: 0.38 – 0.56) and 0.56 (95% CI: 0.37 – 0.84), respectively. In addition, the TMH cohorts were more likely to reside in urban areas, compared to their rural counterparts, with an OR of 0.69 (95% CI: 0.57 – 0.84). Moreover, the TMH cohort was more likely to use commercial insurance or other insurance instead of Medicaid, with ORs of 1.60 (95% CI: 1.31 – 1.96) and 2.21 (95% CI: 1.44 – 3.38), respectively. TMH cohort had a higher odd of patients with a household income exceeding $50,000 instead of less than $42,000 with an OR of 1.81 (95% CI: 1.40 – 2.33).

### HCRU and medical expenditures

Table 2 presents the HCRU and medical expenditures associated with mental and behavioral disorders and overall healthcare. Regarding services related to mental and behavioral health, the TMH cohort demonstrated a significantly higher outpatient visits (mean (SD): 0.589 (0.50) vs 0.239 (0.46) PPPM, *p* < 0.001), inpatient admissions (mean (SD): 0.005 (0.02) vs 0.002 (0.01) PPPM; *p* < 0.001), ED visits (mean (SD): 0.005 (0.02) vs 0.002 (0.01) PPPM, *p* < 0.001), and associated medical expenditures (mean (SD): $47.35 (51.46) vs $21.57 (82.16) PPPM, *p* < 0.001) compared to the non-TMH cohort. Regarding all-cause healthcare services, the TMH cohort had significantly fewer ED visits (mean (SD): 0.045 (0.11) vs 0.057 (0.11) PPPM, *p* = 0.005) and lower overall medical expenditures (mean (SD): $110.97 (159.26) vs $180.82 (261.14) PPPM, *p* < 0.001).

**Table 2:** Mental and behavioral disorder-related and all-cause HCRU and expenditures, PPPM

	Mental and behavioral disorder-related			All-cause		
	TMH (*n* = 793)	Non-TMH (*n* = 1179)	*p* value	TMH (*n* = 793)	Non-TMH (*n* = 1179)	*p* value
Outpatient visits	0.589 (0.50)	0.239 (0.46)	< .001***	1.021 (0.92)	1.186 (1.43)	0.498
Inpatient admissions	0.005 (0.02)	0.002 (0.01)	< .001***	0.011 (0.04)	0.015 (0.05)	0.190
ED visits	0.005 (0.02)	0.002 (0.01)	< .001***	0.045 (0.11)	0.057 (0.11)	0.005**
Medical expenditures, $	47.35 (51.46)	21.57 (82.16)	< .001***	110.97 (159.26)	180.82 (261.14)	< .001***

Abbreviation: *TMH* Tele-Mental Health

*p* values were calculated using the Wilcoxon rank-sum test

^*^ *p* < 0.05, ** *p* < 0.01, *** *p* < .001

Upon adjusting for sociodemographic factors, Table 3 illustrates the estimated TMH effects on HCRU and medical expenditures, both for mental and behavioral health services and overall healthcare. Regarding mental and behavioral health, the TMH cohort displayed 122% higher outpatient visits (*p* < 0.001) and 36% higher medical expenditures (*p* < 0.001) compared to the non-TMH cohort. However, the two cohorts did not significantly differ in the number of inpatient admissions or ED visits. For all-cause healthcare, the TMH cohort had 27% less medical expenditures (*p* < 0.001), but had no significant difference in outpatient visits, inpatient admissions, or ED visits.

**Table 3:** Adjusted TMH effects on mental and behavioral disorder-related HCRU and medical expenditures, PPPM, estimated using GLM (*n* = 1,915)

	Mental and behavioral disorder-related				All-cause	
Outcomes	Estimates (Std Err)	Exponentiated estimates (95% CI)	*p* value	Estimates (Std Err)	Exponentiated estimates (95% CI)	*p* value
Outpatient visits	0.80 (0.08)	2.22 (1.89, 2.62)	< .001***	−0.04 (0.05)	0.96 (0.87, 1.06)	0.438
Inpatient admissions	1.14 (0.93)	3.13 (0.51, 19.40)	0.198	−0.04 (0.46)	0.96 (0.39, 2.37)	0.931
ED visits	0.88 (0.87)	2.42 (0.44, 13.30)	0.296	0.05 (0.23)	1.05 (0.68, 1.64)	0.823
Medical expenditures	0.31 (0.05)	1.36 (1.23, 1.50)	< .001***	-0.32 (0.05)	0.73 (0.66, 0.80)	< .001***

Abbreviations: *TMH* Tele-Mental Health; *HCRU* Health care resource utilization; *PPPM* Per patient per month; *GLM* Generalized linear regression model; *Std Err* Standard error; *CI* Confidence interval

For both mental and behavioral health disorder-related and all-cause outcomes, negative binomial regression models were used for outpatient visits, zero-inflated Poisson regression models were used for inpatient admissions and ED visits, and Gamma regression models were used for medical expenditures

Patient sociodemographic characteristics listed in Table 1 were controlled as covariates in the GLM. Column “Estimates” represents the coefficient estimates of TMH in the GLM model, and column “Exponentiated Estimates” represents the exponential of the coefficient estimates of TMH

^*^ *p* < 0.05, ** *p* < 0.01, *** *p* < .001

## Discussion

### Key findings

This study illuminates the TMH utilization among a pediatric population in Mississippi, revealing notable disparities across sociodemographic groups. Key determinants of TMH utilization include age, race, primary insurance type, rural residency, and household income. This study found that adolescents were more likely to utilize TMH services compared to younger children. Younger children, conversely, at risk for developing mental and behavioral disorders might require specific hands-on clinical assessment, including physical examinations, which cannot be fully met through TMH services.^27,28^

Notably, although UMMC is located in Jackson, where over 80% of the population identified as Black/African American,^29^ this group accounted for less than half of pediatric mental and behavioral health patients and were less likely to utilize TMH services compared to their White/Caucasian counterparts. This discrepancy may point not only to systemic inequalities in healthcare access but also to digital disparities that disproportionately affect underrepresented communities.^30^

Moreover, these digital disparities likely extend to include economic dimensions. Children who were either commercially insured or had other types of insurance, as well as those hailing from households with incomes above $50,000, were more likely to utilize TMH services compared to their less affluent counterparts, specifically those on Medicaid or with household incomes below $42,000. A possible explanation is that families with greater financial resources may have better access to requisite technology and reliable internet connectivity. The higher likelihood of using TMH among urban patients, compared to their rural counterparts, may reflect the unmet healthcare needs among rural residents.

Parents and guardians play an indispensable role in pediatric TMH utilization, given that youth typically rely on their parents to access care.^28^ As such, the sociodemographic characteristics and digital literacy of the family unit have become highly influential factors. Families restricted by limited access to reliable internet or suitable digital devices may find TMH services less accessible, thereby exacerbating existing health disparities. Hence, in alignment with previous studies that highlight the relationship between income and digital disparities,^31^ strategies aimed at increasing TMH adoption should consider patient-centered approaches to avoid exacerbating health inequities.^11,30^

This study further revealed the multifaceted effect of TMH on HCRU and medical expenditures among pediatric patients. Initially, the TMH cohort exhibited notably higher mental and behavioral health associated HCRU and medical expenditures. Conversely, the TMH cohort showed significantly lower overall healthcare services on all fronts. After adjusting for sociodemographic factors, the higher frequency of mental and behavioral health associated outpatient visits and medical expenditures remained statistically significant, as did the lower all-cause medical expenditures. These data indicate that TMH serves as a particularly effective channel for increasing access to outpatient mental and behavioral services, without exacerbating inpatient or ED utilization, underscoring its role in delivering efficient care.

The importance of mental and behavioral health in overall well-being is well-documented.^32–34^ The Centers for Disease Control and Prevention (CDC) has pointed out that youth with poor mental health are at increased risk of drug use, violence, and high-risk sexual behaviors, which can lead to human immunodeficiency virus (HIV), sexually transmitted diseases (STDs), and unintended pregnancies.^35^ Previous research demonstrated the association between outpatient mental and behavioral health services and medical cost savings, predominantly in adult, commercially insured, and diagnosed with cancer cohorts.^21,36,37^ Building on these findings, this study extends this understanding to pediatric patients and suggests that enhanced access to mental and behavioral healthcare via TMH could offer broader benefits, including mitigating associated high-cost health risks and contributing to overall medical cost savings.

Lastly, this study suggests that underlying health disparities may be significant. While unadjusted figures indicated significant differences in inpatient admissions and ED visits between TMH and non-TMH cohorts, these differences diminished after adjusting for sociodemographic factors. This observation underlies the necessity for interventions to ensure equitable healthcare access across sociodemographic groups.

### Limitations

Several limitations should be considered. Firstly, the results are largely confined to the pediatric population receiving care at UMMC, an academic medical center. Consequently, these findings may not fully represent the broader pediatric landscape across Mississippi. Nonetheless, the study offers an important microcosm that sheds light on systemic health inequities. This study aimed to address the gap in literature regarding access to TMH among this population. Future work is warranted to address this gap in practice. Secondly, the potential for missing data on HCRU and medical expenditures is another limitation of selection bias, particularly when patients access mental and behavioral healthcare services from alternative institutions. An attempt was made to mitigate this by focusing on insured patients who consistently sought care from UMMC, but this approach could introduce a bias in the results. Future work should aim to address these limitations by expanding the scope of the study to include a more diverse range of healthcare settings and demographic groups, as well as the diversity in service use patterns.

## Conclusions

This study contributes to the growing body of literature on TMH services, emphasizing not only the sociodemographic determinants that affect its utilization but also its measurable effect on HCRU and medical expenditures among pediatric populations. Importantly, this work identifies the TMH utilization disparities across sociodemographic lines and underscores the pivotal role of TMH in broadening the accessibility of pediatric mental and behavioral healthcare services while contributing to the overall medical cost savings. These data-driven insights can inform a wide spectrum of TMH stakeholders, ranging from clinicians and program implementers to policymakers. Collectively, these findings offer the empirical groundwork for future efforts aimed at ensuring digital equity, thereby fostering the accessibility and affordability in pediatric mental and behavioral healthcare.^38^

## Implications for Behavioral Health

Mental and behavioral health are crucial components of pediatric healthcare, yet access to these services remains limited in rural and underserved areas, leaving many children without diagnosis or treatment. With the growing prevalence of TMH in outpatient care, this study dived into the TMH utilization among a predominantly Black, rural, and Medicaid-enrolled pediatric population in Mississippi. By identifying key sociodemographic factors associated with TMH utilization, the findings of this study can inform targeted interventions aimed at improving access to mental and behavioral healthcare services for underserved children. Additionally, the TMH utilization was identified to be associated with higher numbers of mental and behavioral health-related outpatient visits and medical expenditures, while overall medical expenditures were lower. These findings not only highlight the role of TMH in augmenting outpatient mental healthcare access but also imply the importance of access to mental and behavioral healthcare for overall health status with its potential of cost saving, particularly for this underserved pediatric population.

## Data Availability

Due to the presence of protected health information (PHI) and in accordance with IRB regulations, the datasetcannot be made publicly available.
